# Phase 3, open-label, randomized study comparing 3-monthly with monthly goserelin in pre-menopausal women with estrogen receptor-positive advanced breast cancer

**DOI:** 10.1007/s12282-015-0637-4

**Published:** 2015-09-09

**Authors:** Shinzaburo Noguchi, Hee Jeong Kim, Anita Jesena, Vani Parmar, Nobuaki Sato, Hwei-Chung Wang, Santi Lokejaroenlarb, Jofel Isidro, Ku Sang Kim, Yohji Itoh, Eisei Shin

**Affiliations:** 1 Department of Breast and Endocrine Surgery, Osaka University Graduate School of Medicine, 2-15 Yamadaoka, Suita City, Osaka 565-0871 Japan; 2 Department of Surgery, Asan Medical Center, Seoul, Korea; 3 Cancer Clinic, Iloilo, Philippines; 4 Breast Disease Management Group, Tata Memorial Hospital, Mumbai, India; 5 Department of Breast Oncology, Niigata Cancer Center Hospital, Niigata, Japan; 6 Department of Surgery, China Medical University Hospital, Taichung, Taiwan; 7 Division of General Surgery, Rajavithi Hospital, Bangkok, Thailand; 8 Great Saviour International Hospital, Iloilo, Philippines; 9 Department of Breast and Thyroid Surgery, Ulsan City Hospital, Ulsan, Korea; 10 AstraZeneca K.K., Osaka, Japan

**Keywords:** Goserelin, Pre-menopausal, Advanced breast cancer, Progression-free survival, Estrogen receptor

## Abstract

**Background:**

Monthly goserelin 3.6 mg dosing suppresses estradiol (E2) production and has proven efficacy in pre-menopausal women with estrogen receptor (ER)-positive breast cancer. This non-inferiority study evaluated the efficacy and safety of 3-monthly goserelin 10.8 mg compared with monthly goserelin 3.6 mg.

**Methods:**

This was a Phase 3, open-label, multicenter trial. Pre-menopausal women with ER-positive advanced breast cancer were randomized to 3-monthly goserelin 10.8 mg or monthly goserelin 3.6 mg; all patients received concomitant tamoxifen (20 mg daily). The primary endpoint was progression-free survival (PFS) rate at 24 weeks; non-inferiority was to be confirmed if the entire 95 % confidence interval (CI) for the treatment difference was above −17.5 %. Secondary endpoints included objective response rate (ORR), serum E2 levels, safety, and tolerability.

**Results:**

In total, 222 patients were randomized (goserelin 10.8 mg, *n* = 109; goserelin 3.6 mg, *n* = 113). PFS rate at week 24 was 61.5 % (goserelin 10.8 mg) and 60.2 % (goserelin 3.6 mg); treatment difference (95 % CI) was 1.3 % (−11.4, 13.9), confirming non-inferiority of goserelin 10.8 mg compared with goserelin 3.6 mg. ORR was 23.9 % (goserelin 10.8 mg) and 26.9 % (goserelin 3.6 mg); treatment difference (95 % CI) was −3.0 % (−15.5, 9.7). At week 24, mean serum E2 concentrations were similar in the goserelin 10.8 mg and goserelin 3.6 mg groups (20.3 pg/mL and 24.8 pg/mL, respectively).

**Conclusion:**

A regimen of 3-monthly goserelin 10.8 mg demonstrated non-inferiority compared with monthly goserelin 3.6 mg for PFS rate at 24 weeks, with similar pharmacodynamic and safety profiles, in pre-menopausal women with ER-positive breast cancer.

## Introduction

Approximately two-thirds of patients diagnosed with breast cancer have hormone-receptor–positive [estrogen receptor (ER) and/or progesterone receptor (PgR)] tumors and are suitable candidates for endocrine therapy [1]. Estradiol (E2) is the main source of estrogen in pre-menopausal women and is synthesized and released from the ovaries under the control of luteinising hormone-releasing hormone (LHRH) [2]. Ovarian ablation or suppression can, therefore, be used to impede E2 production, thereby inhibiting estrogen-dependent tumor growth in pre-menopausal patients with ER-positive breast cancer. Goserelin (Zoladex^®^, AstraZeneca) is an LHRH agonist that reduces ovarian E2 production, and, unlike ovarian ablation, its effects are reversible. Goserelin has demonstrated efficacy for the adjuvant treatment of pre-menopausal women with ER-positive breast cancer, with equivalent disease-free survival to cytotoxic chemotherapy [3], and a more favorable safety profile [4].

The efficacy, safety, and endocrine effects of monthly goserelin 3.6 mg, with or without concomitant tamoxifen, in pre- and peri-menopausal women with ER-positive advanced breast cancer are well documented [5–7]. Furthermore, the combination of an LHRH agonist with tamoxifen has been shown to be more effective than each treatment alone [8, 9], and this is now a standard treatment choice in these patients [10, 11].

Goserelin 10.8 mg administered once every 3 months is already approved in many countries for the treatment of prostate cancer [12–14], and this formulation may also provide a more convenient treatment option for breast cancer patients due to its less frequent administration schedule. A recent Phase 2 clinical study found that 3-monthly goserelin 10.8 mg was non-inferior to monthly goserelin 3.6 mg for the primary endpoint of serum E2 suppression in pre-menopausal patients with ER-positive early breast cancer, with no difference in safety profile [15].

The current non-inferiority study was designed to evaluate the efficacy and safety of 3-monthly goserelin 10.8 mg versus monthly goserelin 3.6 mg in pre-menopausal women with ER-positive advanced breast cancer.

## Patients and methods

### Study design and patients

This was a Phase 3, open-label, randomized, parallel group, multicenter trial designed to evaluate whether 3-monthly goserelin 10.8 mg is non-inferior to monthly goserelin 3.6 mg in pre-menopausal women with ER-positive advanced breast cancer by assessment of progression-free survival (PFS) rate at 24 weeks (ClinicalTrials.gov identifier: NCT01073865).

**Fig. 1:**
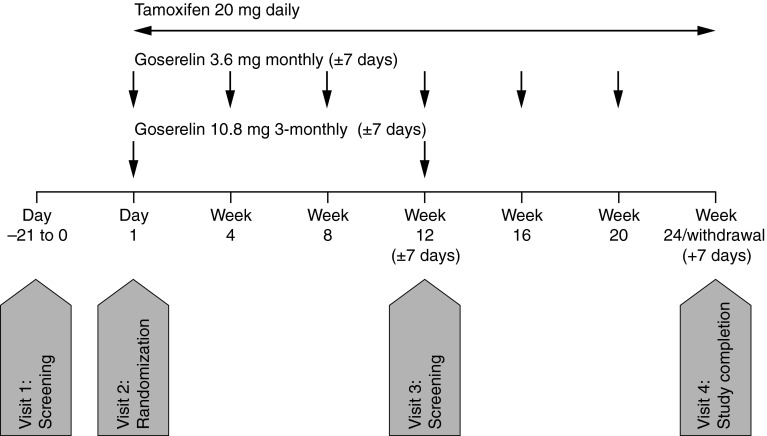
Study design

Eligible patients were randomized 1:1 to receive a goserelin 10.8 mg subcutaneous (sc) depot injection every 12 weeks (±7 days; 3-monthly goserelin 10.8 mg treatment group) or a goserelin 3.6 mg sc depot injection every 4 weeks (±7 days; monthly goserelin 3.6 mg treatment group). All patients also received a daily oral dose of tamoxifen (20 mg) (Fig. 1). The study population comprised pre-menopausal women aged ≥20 years with histologically/cytologically confirmed, hormone-sensitive breast cancer; ≥1 lesion that can be accurately assessed at baseline and is suitable for repeated assessment by CT, MRI, or plain X-ray according to Response Evaluation Criteria In Solid Tumors (RECIST) Version 1.1 [16]; and a World Health Organization performance status of 0, 1, or 2. Pre-menopausal status was defined as experiencing menses within 1 year prior to randomization, and serum concentrations for E2 ≥10 pg/mL and follicle-stimulating hormone (FSH) ≤30 mIU/mL within 4 weeks prior to randomization (for patients who had undergone a hysterectomy, only the latter criterion was required).

Patients were excluded from the study if they had received: hormonal therapies within the previous 24 weeks (or at any point for treatment of advanced breast cancer); LHRH agonist therapy within the previous 48 weeks; radiotherapy or trastuzumab/lapatinib treatment for early breast cancer within the previous 4 weeks (or at any point for treatment of advanced breast cancer); chemotherapy within the previous 4 weeks or for advanced breast cancer (except first-line chemotherapy with the presence of menses and no evidence of disease progression). Additional exclusion criteria included: relapse during or within 48 weeks of adjuvant hormonal therapy, or during or within 24 weeks of adjuvant chemotherapy; previous non-breast cancer malignancy (other than controlled basal/squamous carcinoma or cancer of the cervix); life-threatening metastatic disease; abnormal laboratory tests results; a history of disseminated intravascular coagulation or long-term anticoagulant therapy (other than antiplatelet or warfarin). Patients could not participate if they were likely to be hypersensitive to study treatments, unwilling/unable to stop taking drugs known to affect sex hormone status, were pregnant/breastfeeding, had received an unapproved drug in the previous 12 weeks, were likely to have a survival of <24 weeks, were unlikely to comply with study requirements, or had any concomitant disease that would place them at risk or may confound the results of the study. All patients provided written informed consent.

Patients continued study treatment for 24 weeks or until any of the criteria for discontinuation were met (including voluntary discontinuation, safety issues, medication non-compliance, pregnancy, disease progression, or death). Final assessment of efficacy was completed at the time of disease progression or at week 24, whichever was sooner.

The study protocol was approved by the relevant ethics committees and institutional review boards and was conducted in accordance with the Declaration of Helsinki and the International Conference on Harmonisation/Good Clinical Practice, the applicable local regulatory requirements at each study center, and the AstraZeneca policy on Bioethics. All patients provided written informed consent.

### Efficacy endpoints

The primary endpoint was PFS rate at 24 weeks, used to assess the non-inferiority of 3-monthly goserelin 10.8 mg versus monthly goserelin 3.6 mg. Secondary efficacy endpoints included objective response rate (ORR) and serum E2 concentrations. Efficacy assessments were performed at weeks 12 and 24.

PFS rate, defined as the proportion of patients who were progression-free, was analyzed at 24 weeks. Analysis of ORR, defined as the proportion of patients with a best response of complete or partial response, was determined at each site by RECIST criteria, and assessed in patients who had measurable disease at baseline (e.g., patients with bone metastases). A sub-analysis of efficacy endpoints was carried out to determine if there were differences in treatment response between Japanese and non-Japanese patients. Pharmacodynamic effects were evaluated by recording serum E2 in both treatment groups at baseline and at 12 and 24 weeks. A central laboratory service (Covance Central Laboratory Services) performed chemiluminescent enzyme immunoassay to determine E2 (ADVIA centaur estradiol-6 III, Siemens Medical Solutions Diagnostics, NY, USA) and FSH (Access hFSH, Beckman Coulter Inc., CA, USA) concentrations. The lower limit of quantification (LLOQ) was 18.07 pg/mL for E2, and 0.6 mIU/mL for FSH.

### Safety and tolerability

Assessments of adverse events (AEs) and serious AEs (SAEs) were carried out to compare the safety and tolerability profiles of 3-monthly goserelin 10.8 mg and monthly goserelin 3.6 mg. Final assessment of safety was carried out at week 24 (if treatment was discontinued prior to week 24), 12 weeks after the final dose of goserelin 10.8 mg, or 4 weeks after the final dose of goserelin 3.6 mg. The safety analysis was conducted in all patients who received ≥1 treatment after randomization. A sub-analysis of the incidence of AEs/SAEs was also conducted in Japanese and non-Japanese patients. SAEs were classified by the Medical Dictionary for Regulatory Activities preferred term and system organ class.

### Statistical analysis

Based on a meta-analysis by Klijn et al., the proportion of patients who had not progressed during the first 24 weeks of treatment with the combination of goserelin 3.6 mg and tamoxifen was estimated to be approximately 70 % [9]. Therefore, a sample size of 216 patients (108 per group) was planned to demonstrate non-inferiority based on a pre-specified margin of −17.5 % between treatment groups (deemed clinically acceptable) using a 2-sided 95 % confidence interval (CI) with 80 % power. The non-inferiority margin was chosen based on an admissible 75 % relative efficacy of the 3-monthly goserelin 10.8 mg treatment regimen versus the monthly goserelin 3.6 mg treatment regimen.

Two-sided 95 % CIs were calculated using the score based method recommended by Newcombe and Altman [17]. For the primary endpoint, non-inferiority of the 3-monthly goserelin 10.8 mg treatment regimen versus the monthly goserelin 3.6 mg treatment regimen was to be concluded if the lower limit of the 95 % CI for the difference was greater than −17.5 %. This non-inferiority limit was only applicable to the primary endpoint of PFS at 24 weeks in the overall population; no non-inferiority testing was carried out for other endpoints or subgroups. ORRs at 24 weeks were determined for each treatment, and the difference and 95 % CI numerically compared. Mean serum E2 concentrations were compared using analysis of covariance (ANCOVA), with treatment group, baseline serum E2 concentrations, and ethnicity (Japanese versus non-Japanese) as covariates. Clinical laboratory data were summarized by treatment group using descriptive statistics.

## Results

### Patients

**Fig. 2:**
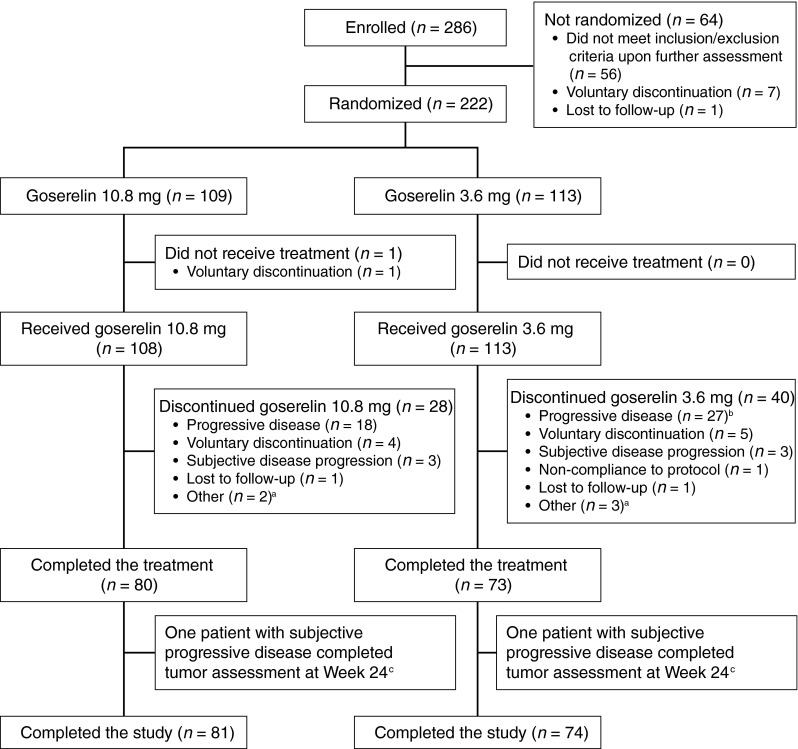
Patient disposition. ^a^ “Other” included death (2 patients in the goserelin 10.8 mg group and 2 patients in the goserelin 3.6 mg group), surgery planned (1 patient in the goserelin 3.6 mg group), and misunderstanding of discontinuation criteria by the patient (1 patient in the goserelin 10.8 mg group). ^b^ Two patients with subjective disease progression who did not meet criteria for progressive disease were incorrectly recorded as having progressive disease. ^c^ Patients who discontinued treatment for any reason other than disease progression were required to continue objective tumor assessments until week 24. If tumor assessment at week 24 was completed, patients were classified as having completed the study

This study was conducted at 58 centers: India (15 centers), Japan (19 centers), Korea (4 centers), the Philippines (7 centers), Thailand (8 centers), and Taiwan (5 centers). In total, 222 patients were randomized to receive 3-monthly goserelin 10.8 mg (*n* = 109) or monthly goserelin 3.6 mg (*n* = 113). Of these patients, 30.6 % (*n* = 68) discontinued from the study [25.7 % (*n* = 28) in the goserelin 10.8 mg group; 35.4 % (*n* = 40) in the goserelin 3.6 mg group] with the most common reason for withdrawal being disease progression (Fig. 2).

All patients were ER-positive, and the majority of patients were both ER- and PgR-positive [82.6 % (*n* = 90) in the goserelin 10.8 mg group; 77.9 % (*n* = 88) in the goserelin 3.6 mg group]. The majority of patients were human epidermal growth factor receptor 2 negative [68.8 % (*n* = 65) in the goserelin 10.8 mg group; 74.3 % (*n* = 84) in the goserelin 3.6 mg group]. Patient demographics and baseline characteristics were well balanced between the treatment groups and also between the treatment groups in the Japanese and non-Japanese subgroups (Table 1).

**Table 1:** Patient demographics and baseline characteristics

	All patients		Japanese patients		Non-Japanese patients		Total (*n* = 222)
	Goserelin 10.8 mg (*n* = 109)	Goserelin 3.6 mg (*n* = 113)	Goserelin 10.8 mg (*n* = 29)	Goserelin 3.6 mg (*n* = 30)	Goserelin 10.8 mg (*n* = 80)	Goserelin 3.6 mg (*n* = 83)	
Median age, years (range)	41.0 (23–53)	42.0 (26–54)	45.0 (27–53)	43.0 (26–54)	40.0 (23–53)	40.0 (27–50)	42.0 (23–54)
Age group, *n* (%)							
<40	42 (38.5)	46 (40.7)	4 (13.8)	8 (26.7)	38 (47.5)	38 (45.8)	88 (39.6)
≥40	67 (61.5)	67 (59.3)	25 (86.2)	22 (73.3)	42 (52.5)	45 (54.2)	134 (60.4)
Ethnicity, *n* (%)							
Asian (non-Chinese/Japanese)	73 (67.0)	74 (65.5)	0	0	73 (91.3)	74 (89.2)	147 (66.2)
Chinese	7 (6.4)	9 (8.0)	0	0	7 (8.8)	9 (10.8)	16 (7.2)
Japanese	29 (26.6)	30 (26.5)	29 (100.0)	30 (100.0)	0	0	59 (26.6)
Previous type of therapy,^a^ *n* (%)							
Radiotherapy	19 (17.4)	17 (15.0)	4 (13.8)	2 (6.7)	15 (18.8)	15 (18.1)	36 (16.2)
Chemotherapy	29 (26.6)	27 (23.9)	3 (10.3)	2 (6.7)	26 (32.5)	25 (30.1)	56 (25.2)
Other systemic anticancer therapy	1 (0.9)	0	0	0	1 (1.3)	0	1 (0.5)
Immunotherapy	0	0	0	0	0	0	0
Hormonal therapy	9 (8.3)	10 (8.8)	4 (13.8)	1 (3.3)	5 (6.3)	9 (10.8)	19 (8.6)
WHO performance status, *n* (%)							
0	83 (76.1)	92 (81.4)	22 (75.9)	26 (86.7)	61 (76.3)	66 (79.5)	175 (78.8)
1	26 (23.9)	18 (15.9)	7 (24.1)	4 (13.3)	19 (23.8)	14 (16.9)	44 (19.8)
2	0	3 (2.7)	0	0	0	3 (3.6)	3 (1.4)
Disease stage, *n* (%)							
Locally advanced only	9 (8.3)	16 (14.2)	3 (10.3)	4 (13.3)	6 (7.5)	12 (14.5)	25 (11.3)
Metastatic	100 (91.7)	97 (85.8)	26 (89.7)	26 (86.7)	74 (92.5)	71 (85.5)	197 (88.7)
Measurable disease, *n* (%)							
Yes	88 (80.7)	93 (82.3)	27 (93.1)	27 (90.0)	61 (76.3)	66 (79.5)	181 (81.5)
No	21 (19.3)	20 (17.7)	2 (6.9)	3 (10.0)	19 (23.8)	17 (20.5)	41 (18.5)

*WHO* World Health Organization

^a^Details for previous therapies for one patient were not recorded in the database

### Efficacy

**Fig. 3:**
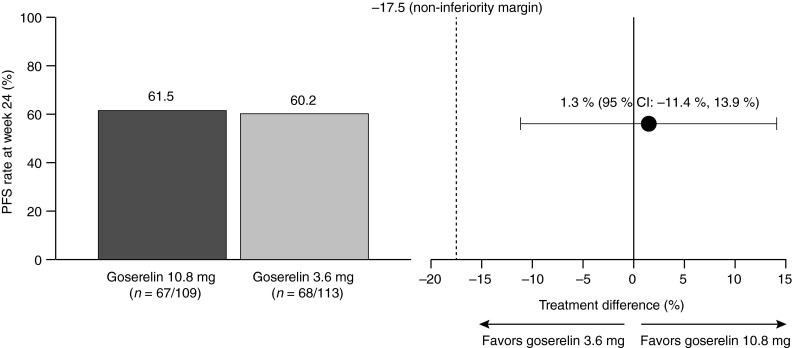
Progression-free survival at week 24: non-inferiority analysis. *CI* confidence interval, *PFS* progression-free survival

In total, 61.5 % (*n* = 67) of patients in the goserelin 10.8 mg group and 60.2 % (*n* = 68) of patients in the goserelin 3.6 mg group were progression-free at 24 weeks (treatment difference: 1.3; 95 % CI −11.4, 13.9). Since the lower 95 % CI was above the pre-defined margin of −17.5 %, 3-monthly goserelin 10.8 mg met the criteria for non-inferiority compared with monthly goserelin 3.6 mg (Fig. 3).

In the Japanese population, 72.4 % (*n* = 21/29) of patients in the goserelin 10.8 mg group and 80.0 % (*n* = 24/30) of patients in the goserelin 3.6 mg group were progression-free at 24 weeks (treatment difference: −7.6; 95 % CI −28.5, 14.0). In the non-Japanese population, 57.5 % (*n* = 46/80) of patients in the goserelin 10.8 mg group and 53.0 % (*n* = 44/83) of patients in the goserelin 3.6 mg group were progression-free at 24 weeks (treatment difference: 4.5; 95 % CI −10.6, 19.4).

At 24 weeks, the ORR in patients with measurable disease at baseline was 23.9 % (*n* = 21/88) in the goserelin 10.8 mg group and 26.9 % (*n* = 25/93) in the goserelin 3.6 mg group (treatment difference: −3.0 %; 95 % CI −15.5, 9.7; Table 2). In Japanese patients, ORR was 25.9 % (*n* = 7/27) in the goserelin 10.8 mg group and 37.0 % (*n* = 10/27) in the goserelin 3.6 mg group (treatment difference: −11.1 %; 95 % CI −33.8, 13.2). In non-Japanese patients the ORR was 23.0 % (*n* = 14/61) in the goserelin 10.8 mg group and 22.7 % (*n* = 15/66) in the goserelin 3.6 mg group (treatment difference: 0.2 %; 95 % CI −14.2, 14.9).

**Table 2:** Best objective response at week 24

	Goserelin 10.8 mg (*n* = 88)^a^	Goserelin 3.6 mg (*n* = 93)^a^
Complete response, *n* (%)	1 (1.1)	0
Partial response, *n* (%)	20 (22.7)	25 (26.9)
ORR, *n* (%)	21 (23.9)	25 (26.9)
Treatment difference, % (95 % CI)	−3.0 (−15.5, 9.7)	

*CI* confidence interval, *ORR* objective response rate

^a^Included only patients who had measurable disease at baseline

### Pharmacodynamics

**Fig. 4:**
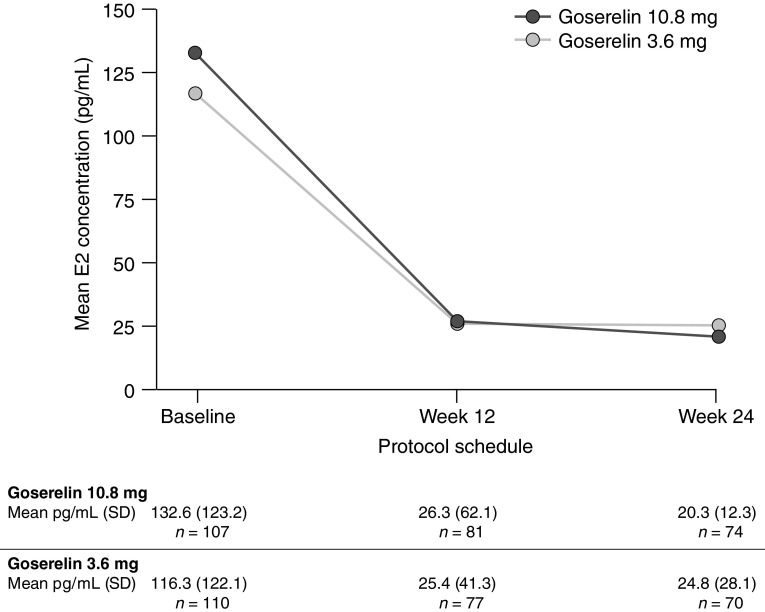
Serum E2 concentration at baseline, 12 weeks and 24 weeks. *E2* estradiol, *SD* standard deviation

Mean (standard deviation) serum E2 concentrations throughout the study period are presented in Fig. 4. At week 24, serum E2 concentrations were 20.3 (12.3) pg/mL and 24.8 (28.1) pg/mL in the goserelin 10.8 mg and goserelin 3.6 mg groups, respectively.

### Safety and tolerability

A total of 143 of 221 (64.7 %) patients reported ≥1 AE [65.7 % (*n* = 71) of patients in the goserelin 10.8 mg group and 63.7 % of patients (*n* = 72) in the goserelin 3.6 mg group]. The most common AEs are shown in Table 3; the majority of AEs were mild or moderate (Grade 2 or lower).

**Table 3:** Common AEs occurring in ≥3 % of patients

AE by system organ class	Goserelin 10.8 mg (*n* = 108)	Goserelin 3.6 mg (*n* = 113)	Total (*n* = 221)
Patients with any AE, *n* (%)	71 (65.7)	72 (63.7)	143 (64.7)
Vascular disorders, *n* (%)			
Hot flush	15 (13.9)	22 (19.5)	37 (16.7)
Infections and infestations, *n* (%)			
Nasopharyngitis	13 (12.0)	9 (8.0)	22 (10.0)
Musculoskeletal and connective tissue disorders, *n* (%)			
Back pain	5 (4.6)	9 (8.0)	14 (6.3)
Pain in extremity	3 (2.8)	5 (4.4)	8 (3.6)
Nervous system disorders, *n* (%)			
Headache	7 (6.5)	7 (6.2)	14 (6.3)
Gastrointestinal disorders, *n* (%)			
Nausea	2 (1.9)	9 (8.0)	11 (5.0)
Constipation	4 (3.7)	5 (4.4)	9 (4.1)
Vomiting	4 (3.7)	3 (2.7)	7 (3.2)
Abdominal pain	1 (0.9)	4 (3.5)	5 (2.3)
General disorders and administration site conditions, *n* (%)			
Pyrexia	2 (1.9)	7 (6.2)	9 (4.1)
Fatigue	1 (0.9)	4 (3.5)	5 (2.3)
Psychiatric disorders, *n* (%)			
Insomnia	3 (2.8)	6 (5.3)	9 (4.1)
Respiratory, thoracic and mediastinal disorders, *n* (%)			
Cough	3 (2.8)	4 (3.5)	7 (3.2)
Blood and lymphatic system disorders, *n* (%)			
Anemia	4 (3.7)	4 (3.5)	8 (3.6)

*AE* adverse event

A total of 12 patients reported a SAE (4 patients in the goserelin 10.8 mg group and 8 patients in the goserelin 3.6 mg group). The incidence of SAEs was 3.7 % in the goserelin 10.8 mg group and 7.1 % in the goserelin 3.6 mg group. A total of 7 patients died in the study (4 patients in goserelin 10.8 mg group, 3 patients in goserelin 3.6 mg group). Three patients in the goserelin 10.8 mg group and 2 patients in the goserelin 3.6 mg group died due to disease progression. One patient in each group died following an AE. One patient was a 23-year-old female who had breast lumpectomy approximately 4 months prior to receiving goserelin 10.8 mg and had received zoledronic acid due to bone metastasis with no other prior medications or radiotherapy. This patient had been judged to have stable disease 2 months after their first depot 10.8 mg goserelin dose; 2 months after the second dose this patient reported chest pain, diarrhea and vomiting, and died. The other patient was a 34-year-old female who had previously had modified radical mastectomy of the right breast and had received adjuvant tamoxifen and chemotherapy (5-fluorouracil, epirubicin and cyclophosphamide) approximately 2.5 years prior to receiving goserelin. Approximately 3 months after the first depot goserelin 3.6 mg dose, this patient had been judged to have partial disease response. Approximately 2 weeks after the 6th dose, the patient developed dyspnea and pyrexia and died due to dyspnea. Neither of these deaths were judged by the investigators to be related to study treatment.

In Japanese patients, the incidence of AEs was 96.6 % (*n* = 28/29) in the goserelin 10.8 mg group and 83.3 % (*n* = 25/30) in the goserelin 3.6 mg group. Two SAEs occurred among Japanese patients, both in the goserelin 3.6 mg group. In non-Japanese patients, the incidence of AEs was 54.4 % (*n* = 43/79) in the goserelin 10.8 mg group and 56.6 % (*n* = 47/83) in the goserelin 3.6 mg group. SAEs were experienced by 5.1 % (*n* = 4/79) of non-Japanese patients in the goserelin 10.8 mg group and 7.2 % (*n* = 6/83) in the goserelin 3.6 mg group.

## Discussion

This randomized, open-label, Phase 3 controlled trial compared PFS rate in pre-menopausal women with ER-positive advanced breast cancer after 24 weeks of treatment with 3-monthly goserelin 10.8 mg or monthly goserelin 3.6 mg. The result of the primary efficacy analysis found that goserelin 10.8 mg demonstrated non-inferiority to goserelin 3.6 mg. Secondary outcomes, such as ORR, were also similar between both treatment groups, suggesting that the similarities seen between the 2 dosing regimens are consistent across several efficacy parameters. PFS rate and ORR were both numerically higher in the Japanese subgroup; however, the sample sizes for these analyses were too small to enable definitive conclusions to be drawn.

Results from the present study demonstrate that the goserelin 10.8 mg group elicited levels of serum E2 suppression at week 24 that were comparable with those in the goserelin 3.6 mg group. These results are similar to those reported in another clinical study comparing 3-monthly goserelin 10.8 mg with monthly goserelin 3.6 mg treatment in pre-menopausal women with early-stage breast cancer, in which serum E2 concentrations were also suppressed to post-menopausal levels (≤30 pg/mL) in both treatment groups [15]. There were no obvious differences in suppression of serum E2 concentration between Japanese and non-Japanese patients. Goserelin 10.8 mg was well tolerated and showed a similar safety and tolerability profile to goserelin 3.6 mg. Treatment-related AEs reported in this study were consistent with the established safety profile for goserelin 10.8 mg (given for the treatment of prostate cancer) and goserelin 3.6 mg in female patients. The incidence of AEs appeared to be higher in Japanese than in non-Japanese patients; the reason for this is unclear. However, the incidence of SAEs was similarly low in both Japanese and non-Japanese patients. In addition, there were no clear differences in the incidence of AEs between goserelin 10.8 mg and goserelin 3.6 mg treatment groups when assessing the Japanese and non-Japanese subgroups.

Anecdotal evidence suggests that monthly sc depot injections are uncomfortable for the patient [18]. A dosing regimen of 3-monthly goserelin 10.8 mg offers an alternative dosing schedule that may be more convenient for some patients, thus, potentially helping to improve compliance. Moreover, a 3-month dosing regimen with fewer clinic visits could help to reduce clinician burden and associated healthcare costs.

Potential limitations of the study include the open-label design and the relatively short study length. The expected difficulty of enrolling patients into the study from a limited population pool (i.e., pre-menopausal patients with advanced breast cancer) was taken into consideration when designing the study. As goserelin was used in combination with tamoxifen in both treatment groups, it was also not possible to fully determine the effect of the individual treatments (tamoxifen or goserelin) used in the study. However, this combination therapy approach was in line with current clinical practice guidelines [10, 19].

In conclusion, this study demonstrated that 3-monthly goserelin 10.8 mg is non-inferior to monthly goserelin 3.6 mg in pre-menopausal women with ER-positive advanced breast cancer by assessment of PFS rate at 24 weeks. As such, this formulation may represent an alternative and more convenient treatment option for pre-menopausal women with ER-positive advanced breast cancer.
